# A multistrain probiotic formulation attenuates skin symptoms of atopic dermatitis in a mouse model through the generation of CD4^+^Foxp3^+^ T cells

**DOI:** 10.3402/fnr.v60.32550

**Published:** 2016-10-31

**Authors:** Joo-Hyun Shin, Myung-Jun Chung, Jae-Gu Seo

**Affiliations:** R&D Center, Cell Biotech Co., Ltd., Gyeonggi-do, Republic of Korea

**Keywords:** atopic dermatitis, inflammation, probiotics, intestinal microbiota, regulatory T cells

## Abstract

**Background:**

Atopic dermatitis (AD) is characterized by chronic inflammation of the skin. AD develops mainly in infants and young children. It induces skin disorders and signals the initiation of the allergic march including allergic asthma and rhinitis. Probiotics modify intestinal microbial populations in a beneficial way for human and animal hosts by reducing inflammatory cytokines.

**Objective:**

As a result of their immunomodulatory properties, probiotics have been considered a promising therapeutic option for the prevention and treatment of AD.

**Design:**

In this study, we examined the effects of GI7, a potential probiotic mixture consisting of seven strains of bifidobacteria and lactic acid bacteria, on AD in a mouse model.

**Results:**

Administration of GI7 for 8 weeks reduced AD-like skin lesions and induced changes in the levels of serum markers such as immunoglobulin E and cytokines related to T helper (Th)1 and Th2 cells, and in skin barrier genes. Alleviation of AD seems to be associated with GI7-induced generation of CD4^+^Foxp3^+^ regulatory T cells.

**Conclusions:**

The probiotic mixture may have potential to improve symptoms of AD.

Atopic dermatitis (AD) is a common chronic inflammatory skin disease that has dramatically increased in prevalence over the past several decades, particularly in industrialized countries (1). Although the pathophysiology of AD is not yet fully understood, it is known to have complicated interactions with environmental and genetic factors that increase abnormalities in the structure and barrier function of skin, and in the immune system (2, 3). AD skin lesions contain high levels of mast cells, eosinophils, and T helper (Th)2 cells and display historical abnormalities including hyperkeratosis and hyperplasia (4). The cytokine milieu of AD skin lesions show Th2-dominant responses indicated by the excessive production of thymus- and activation-regulated chemokine (TARC) and interleukin (IL)-4, IL-5, and IL-13 that leads to an immune response resulting in elevated serum immunoglobulin E (IgE) levels (5). These responses are caused by thymic stromal lymphopoietin (TSLP) produced by keratinocytes (KCs) (6). Although Th2 cells are dominant during the acute phase of AD, interferon (IFN)-γ- and IL-12–producing Th1 cells are expressed and contribute to the pathogenesis during the chronic phase (7). AD skin lesions show prominent infiltrates of mononuclear cells in the dermis combined with intercellular edema in the epidermis and impairment of skin barrier function. The permeability barrier function is not only regulated by corneocytes but also by the stratum corneum lipid-enriched extracellular matrix that mainly consists of the structural proteins loricrin (Lor), involucrin (Ivl), and filaggrin (Flg) and small proline-rich proteins (8). Transglutaminase (Tgm) enzymes are also similarly involved in the epidermal barrier formation.

Probiotics are noninvasive, nonpathogenic microorganisms known for promoting human health. Probiotics modulate the indigenous gastrointestinal (GI) microbiota and immune reactions through multiple mechanisms including the direct inhibition of enteric pathogen activity by lowering luminal pH, the secretion of bacterial proteins, the induction of the epithelial defense mechanism, and modification of immunoregulation by decreasing proinflammatory molecules and promoting protective molecules (9). The mechanism of action of probiotics still remains only partially understood. In a previous report, probiotics were shown to promote immune tolerance by acting on dendritic cells (DCs); promoting regulatory T cell (Treg) development; and/or decreasing Th1, Th2, and Th17 cell responses in an AD mouse model (10). Toll-like receptor (TLR) activation seems to be crucial for the immunomodulatory effect of probiotics.

Expression of forkhead box P3 (Foxp3), a key regulator of Treg development, is known as a specific CD4^+^ Treg marker (11). CD4^+^Foxp3^+^ Tregs can suppress the activation or proliferation of various immune cells, including Th1, Th2, and Th17 cells. A previous study has reported that administration of a probiotic mixture including five probiotics strains, *Bifidobacterium lactis*, *Lactobacillus acidophilus*, *Lactobacillus casei*, *Lactobacillus reuteri*, and *Streptococcus thermophilus*, showed therapeutic effects in experimental inflammatory bowel disease (IBD), AD, and rheumatoid arthritis (RA) (10). The therapeutic effects were found to be related with the generation of CD4^+^Foxp3^+^ Tregs in the inflamed regions. In addition, mutations in Foxp3 have been shown to induce immunodysregulation, polyendocrinopathy, enteropathy, and X-linked syndrome (12, 13).

Recently, we formulated a multispecies probiotic mixture named GI7. This blend contains seven bacterial strains: *Bifidobacterium bifidium* CBT BF3, *Bifidobacterium breve* CBT BR3, *L. acidophilus* CBT LA1, *Lactobacillus plantarum* CBT LP3, *Lactobacillus rhamnosus* CBT LR5, *Lactococcus lactis* CBT SL6, and *Streptococcus thermophilus* CBT ST3. These strains were selected based on their anti-inflammatory activity determined in lipopolysaccharide (LPS)-treated RAW254.7 cells (Kim et al., manuscript in preparation). The anti-inflammatory activity of the GI7 combination seemed to be superior to those of the individual strains, and the administration of GI7 suppressed the progression of experimental colitis. Thus, the results support its anti-inflammatory potency. In this study, we investigated whether GI7 has potential therapeutic effects on AD-like skin lesions by using a 2,4-dinitrochlorobenzene (DNCB)–induced mouse model. Because NC/Nga mice, which have genetically reduced skin barrier function, develop severe and persistent AD, this strain of mouse was used in our study (14). Activation of Foxp3^+^ Tregs, serum IgE, and inflammatory cytokine levels; skin barrier– and antimicrobial-related gene and chemokine expression; and fecal microbiota were also examined.

## Materials and methods

### Preparation of bacterial mixture

GI7 (Cell Biotech, Co., Ltd, Gimpo, Korea) is a multispecies probiotic mixture containing seven species of bacteria: *Lactobacillus acidophilus* CBT LA1 (KCTC 11906BP), *Lactobacillus rhamnosus* CBT LR5 (KCTC 12202BP), *Lactobacillus plantarum* CBT LP3 (KCTC 10782BP), *Bifidobacterium bifidum* CBT BF3 (KCTC 12199BP), *Bifidobacterium breve* CBT BR3 (KCTC 12201BP), *Lactococcus lactis* CBT SL6 (KCTC 111865BP), and *Streptococcus thermophilus* CBT ST3 (KCTC 11870BP). The same viable cell counts of each strain and excipients (i.e. dextrose, maltodextrin, microcrystalline-cellulose, and Mg-stearate) were mixed to produce a dry powder.

### Cell culture and treatment

LPS from *Escherichia coli* (O55:B5) was purchased from Sigma (St. Louis, MO, USA). The mouse macrophage cell line RAW264.7 was cultured in Dulbecco's modified Eagle's medium supplemented with 10% fetal bovine serum (FBS) and 1% penicillin streptomycin. Cells were cultured in six-well plates with 15 ng/mL LPS for 6 h with or without individual bacterial strains (10^7^ colony-forming units [CFU]/mL). For treatment with GI7, the seven strains were separately cultivated overnight at 37°C and collected by centrifugation. Each strain was mixed equally to produce 10^7^ CFU/mL.

### Animals

Male NC/Nga mice (5 weeks old, 18–20 g) were obtained from Saeronbio Inc. (Uiwang, Korea). All animals were housed in standard plastic cages (four mice per cage) and maintained under a 12-h light/dark cycle at constant temperature (23±1°C) and humidity (55–65%) with free access to food and water. The use and care of animals were reviewed and approved by the Institutional Animals Care and Use Committee at the Cell Biotech R&D center (CBTJ-15-02), and all animal procedures were in accordance with the Guide for the Care and Use of Laboratory Animals issued by the Laboratory Animal Resources Commission of Cell Biotech R&D center.

### Atopic dermatitis model

**Fig. 1:**
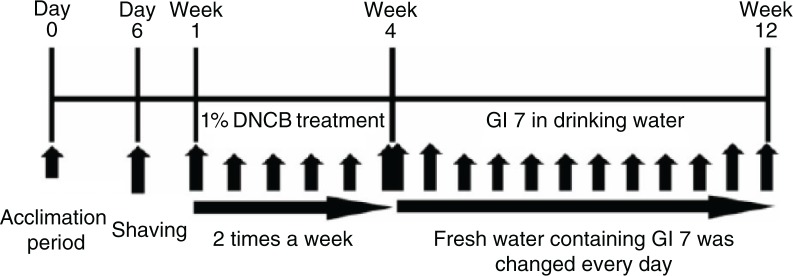
Experimental design. NC/Nga mice were divided into four groups: (1) normal control (Control), (2) DNCB+excipient (Excipient), (3) DNCB+GI7 (10^7^ CFU/mL) (GI7-L), and (4) DNCB+GI7 (10^9^ CFU/mL) (GI7-H) (*n*=12 per group). To study the effect of GI7 on atopic dermatitis, mice were treated with DNCB for 3 weeks and excipient- or GI7-containing water was fed for 8 weeks after the DNCB treatment.

After acclimatization for 1 week (AD-like skin lesions were induced in 6-week-old male NC/Nga mice by using 1-chloro-2,4-dinitrobenzene (DNCB, Sigma, St. Louis, USA)). In brief, the dorsal hair was shaved using an electronic clipper 1 day before DNCB treatment. The DNCB solution was prepared at a concentration of 1% in an acetone:olive oil suspension (3:1), and repeated challenge was performed on the dorsal skin of mice twice a week for 3 weeks. The mice were divided into the following four groups (*n*=12 per group): normal control (control), DNCB with the excipient of GI7 (excipient), DNCB with GI7 (10^7^ CFU/day, GI7-L), and DNCB with GI7 (10^9^ CFU/day, GI7-H). Mice were fed excipient or GI7 in their drinking water every day for 8 weeks after 3 weeks of DNCB treatment. During the GI7 administration, mice were challenged with 0.5% DNCB once a week (Fig. 1).

### Evaluation of skin lesion

The severity of dermatitis score was evaluated once a week after 3 weeks of DNCB treatment. Scores of 0 (none), 1 (mild), 2 (moderate), and 3 (severe) were measured for each of the four symptoms: 1) erythema/hemorrhage, 2) scarring/dryness, 3) edema, and 4) excoriation/erosion. The sum of the individual scores indicating clinical severity was taken as the dermatitis score.

### Evaluation of scratching behavior

AD-like behavioral change was evaluated by measuring the time that mice spent scratching their nose, ears, and dorsal skin with their paws for 10 min on the last day of the experiment (at day 84).

### RNA isolation and qPCR

Samples from dorsal lesioned skin were collected, frozen instantly in liquid nitrogen (LN), and stored at −80°C. Samples were homogenized by pounding in a mortar in LN. For RNA isolation from the skin samples and RAW264.7 cells, the NucleoSpin^®^ RNA purification kit (Macherey-Nagel, Düren, Germany) was used according to the manufacturer's protocol. RNA was reverse transcribed with HyperScript reverse transcription reagents (GeneAll Biotechnology, Seoul, Korea), and quantitative PCR (qPCR) was performed with the SYBR Green Supermix (iQ SYBR Green Supermix, Bio-Rad Laboratories, Hercules, CA, USA) on the LightCycler^®^ 480 Real-Time PCR System (Roche, Indianapolis, IN, USA). The mRNA levels of target genes were quantified relative to that of the reference gene GAPDH. Relative gene expression was analyzed by the comparative ΔC_T_ method as described by Schmittgen and Livak (15). The PCR primers used for gene analysis are listed in Table 1, and all PCR conditions were as per those used in the corresponding studies cited.

**Table 1:** qPCR primer sequences for gene analysis

Gene	Primer sequences	Reference
GAPDH	F: 5′-AAC TTT GGC ATT GTG GAA GG-3′	(43)
	R: 5′-ACA CAT TGG GGG AGG AAC A-3′	
TNF-α	F: 5′-CAG ACC CTC ACA CTC AGA TCA TCT-3′	(44)
	R: 5′-CCT CCA CTT GGT GGT TTG CTA-3′	
IL-1β	F: 5′-AAG GGC TGC TTC CAA ACC TTT GAC-3′	(44)
	R: 5′-ATA CTG CCT GCC TGA AGC TCT TGT-3′	
TSLP	F: 5′-AGA GAA GCC CTC AAT GAC CA T-3′	(45)
	R: 5′-GGA CTT CTG TGC CAT TTC C-3′	
TARC	F: 5′-CAG GAA GTT GGT GAG CTG GTA TA-3′	(46)
	R: 5′-TTG TGT TCG CCT GTA GTG CAT A-3′	
Lor	F: 5′-TCC TTC CCT CAC TCA TCT TCC-3′	(45)
	R: 5′-CTC CTC CAC CAG AGG TCT TTC-3′	
Ivl	F: 5′-CTC CTG TGA GTT TGT TTG GTC T-3′	(45)
	R: 5′-GGA TGT GGA GTT GGT TGC TT-3′	
Tgm1	F: 5′-AGA CCC AAG GTC CTC AAT GTC-3′	(45)
	R: 5′-ACT TGG GAA AGC TGT GGA CTG-3′	
Flg	F: 5’-CAC TGA GCA AAG AAG AGC TGA A-3′	(45)
	R: 5′-CGA TGT CTT GGT CAT CTG GA-3′	
Defb3	F: 5′-CTC CAC CTG CAG CTT TTA GC-3′	(45)
	R: 5′-GGA ACT CCA CAA CTG CCA AT-3′	
Sema3A	F: 5′-AGA TGC TCC ATT CCA GTT TGT TCA C-3′	(47)
	R: 5′-ACA TAA GCC ACC GCA TCA CTT GTA-3′	
Foxp3	F: 5′-CCC ATC CCC AGG AGT CTT G-3′	(16)
	R: 5’-CCA TGA CTA GGG GCA CTG TA-3′	

### Histological examination

To evaluate epidermal thickening, the dorsal lesioned skin of each mouse was fixed with 10% neutral-buffered formalin and embedded in paraffin on the last day of the experiment (at week 12). Then, 4-µm-thick sections were cut and transferred onto slides, and deparaffinized skin sections were stained with hematoxylin and eosin (H&E). For histological grading, epithelial hypertrophy and hyperkeratosis of each mouse were scored as follows: 0, normal thickness; 1, two times normal thickness; 2, three times normal thickness; 3, four times normal thickness; or 4, greater than four times normal thickness. To detect eosinophil and mast cell infiltration, the dorsal lesioned skin of each mouse was also stained with Congo red (CR) and toluidine blue (TB), respectively. The number of eosinophils and mast cells was counted at 200× magnification under a florescence microscope (BX53, Olympus, Tokyo, Japan) in three random areas.

### Flow cytometry analysis

Single-cell suspensions were prepared from the mesenteric lymph nodes (MLNs) of each group. CD4^+^T cells were isolated by using CD4^+^T cell isolation beads and columns (Miltenyi Biotec, Auburn, CA, USA) and labeled with fluorescein isothiocyanate anti-mouse CD4 monoclonal antibody (mAb) (BioLegend, San Diego, CA, USA) and APC anti-mouse CD25 mAb (BioLegend, San Diego, CA, USA). To detect Foxp3, cells were fixed and permeabilized with Foxp3 staining buffer (eBioscience, San Diego, CA, USA) and labeled with anti-mouse/rat Foxp3 PE (eBioscience, San Diego, CA, USA). Flow cytometry was performed using the MACSQuant Analyzer 10 (Miltenyi Biotec, Auburn, CA, USA), and data were analyzed using MACSQuantify software (Miltenyi Biotec, Auburn, CA, USA).

### Measurement of serum IgE levels and multiplex bead-based immunoassay

At the end of the 8-week treatment period, the mice were anesthetized with Zoletil (30 mg/kg body weight). Blood samples were collected by heart puncture into heparinized tubes, and the serum was collected by centrifugation at 2,000*g* for 20 min at 4°C. IgE levels were determined using a mouse IgE ELISA kit (BD Biosciences, Franklin Lakes, NJ, USA). In brief, a 1:250 dilution of anti-mouse IgE monoclonal antibody in phosphate-buffered saline (PBS) was placed in an immunoplate (Nunc A/S, Roskilde, Denmark) and maintained overnight at 4°C. After washing wells three times with PBS containing 0.05% Tween 20 (washing buffer), 200 µL of PBS containing 10% FBS was placed in each well. After incubation for 1 h at room temperature, wells were washed three times with washing buffer, and serum samples (100 µL) were then placed in the wells. The serum samples were first diluted with PBS containing 10% FBS. After a 2-h incubation at room temperature, wells were washed three times with washing buffer, and streptavidin-horseradish peroxide–conjugated detection antibody was added. After further incubation for 1 h at room temperature, wells were washed five times with washing buffer, and the enzyme reaction was initiated by addition of 100 µL of substrate solution (0.1 M citric acid, 0.M Na_2_HPO_4_, *o*-phenylene diamine, and H_2_O_2_). After 20 min at room temperature, the reactions were terminated by the addition of 50 µL of 2 N H_2_SO_4_ to each well, and the absorbance of each well was immediately measured at 450 nm by using an ELISA reader (Bio-Rad Laboratories, Hercules, CA, USA). Simultaneous assessment of serum concentrations of IL-4, IL-10, IL-13, IL-12p40, and IFN-γ was performed using multiplex bead-based sandwich immunoassay kits (MCYTOMAG-70K-05, Millipore, Billerica, MA, USA) according to the manufacturer's instructions. In brief, serum samples (25 µL per well) or standards (25 µl per well) were mixed with 25 µl of the premixed bead sets and incubated in prewetted 96-well microtiter plates overnight at 4°C. After washing, fluorescent detection antibody mixture was added for 30 min, followed by the addition of streptavidin-phycoerythrin for 30 min at room temperature. Cytokine concentrations were determined using a Luminex 100 IS analyzer (Luminex, Austin, TX, USA), and the data were analyzed as median fluorescent intensities.

### Fecal microbiota analysis

Stool samples were collected at the end of the study and were frozen and stored at −20°C until analysis. For quantification of a few groups of bacteria, DNA was extracted from 0.1 g of feces by using the FastDNA™ spin kit (MP Biomedicals, Solon, OH, USA) according to the manufacturer's protocol. DNA concentration and quality were assessed spectrometrically. qPCR was performed using SYBR Green Supermix (Bio-Rad Laboratories, Hercules, CA, USA) on LightCycler 480 Real-Time PCR System (Roche, Indianapolis, IN, USA). The PCR primers used for fecal microbiota analyses are listed in Table 2, and all PCR conditions were as per those used in the corresponding studies cited.

**Table 2:** qPCR primer sequences for fecal microbiota analysis

Target	Primer sequences	Reference
Total bacteria	F: 5′- CCT ACG GGA GGC AGC AG-3′	(48)
	R: 5′-ATT ACC GCG GCT GCT GG-3′	
*Lactobacillus acidophilus*	F: 5′-GAA AGA GCC CAA ACC AAG TGA TT-3′	(48)
	R: 5′-CTT CCC AGA TAA TTC AAC TAT CGC TTA-3′	
*Lactobacillus rhamnosus*	F: 5′-CTA GCG GGT GCG ACT TTG TT-3′	(48)
	R: 5′-GCG ATG CGA ATT TCT ATT AT-3′	
*Lactobacillus plantarum*	F: 5′-ATT CAT AGT CTA GTT GGA GGT-3′	(48)
	R: 5′-CCT GAA CTG AGA GAA TTT GA-3′	
*Bifidobacterium bifidum*	F: 5′- CCA CAT GAT CGC ATG TGA TTG-3′	(49)
	R: 5′-CCG TTG GCT TGC TCC CAA A-3′	
*Bifidobacterium breve*	F: 5′-CCG GAT GCT CCA TCA CAC-3′	(49)
	R: 5′-ACA AAG TGC CTT GCT CCC T-3′	
*Lactococcus lactis*	F: 5′- GCA ATT GCA TCA CTC AAA GA-3′	(50)
	R: 5′-ACA GAG AAC TA TAG CTC CC-3′	
*Streptococcus thermophilus*	F: 5′-ACG GAA TGT ACT TGA GTT TC-3′	(48)
	R: 5′-TTT GGC CTT TCG ACC TAA C-3′	

### Statistical analysis

Results were expressed as mean±standard deviation (SD). Statistical analysis was performed using analysis of variance and Tukey's multiple-range tests. Differences were considered to be significant if *p*<0.05.

## Results

### GI7 alleviated DNCB-induced AD-like symptoms in NC/Nga mice

**Fig. 2:**
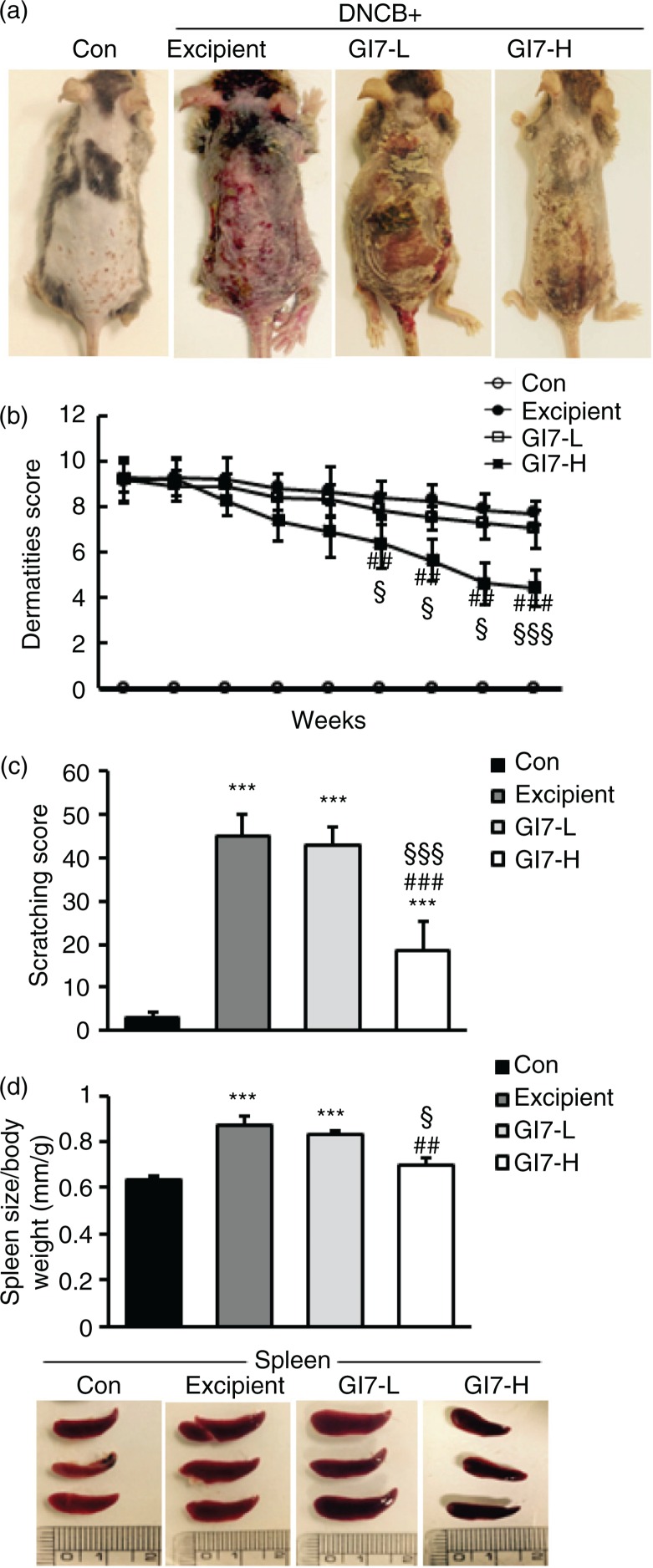
Effect of GI7 on DNCB-induced AD-like symptoms in NC/Nga mice. (a) DNCB-induced AD-like skin lesions in NC/Nga mice. (b) The dermatitis score of AD-like skin lesions was measured concerning itching, erythema/hemorrhage, scaling/dryness, edema, and excoriation/erosion every week. (c) Scratching behavior of the mice was observed for 10 min after sensitization. (d) After sacrifice, the spleen was isolated and photographed to examine morphological alteration. Spleen size-to-body weight ratio was evaluated. Results are expressed as the means±SD (*n*=12). ***p<0.001 versus the control group; ^##^ *p*<0.01, ^###^ *p*<0.001 versus the excipient group; ^§^ *p*<0.05, ^§§§^ *p*<0.001 versus the GI7-L group.

**Fig. 3:**
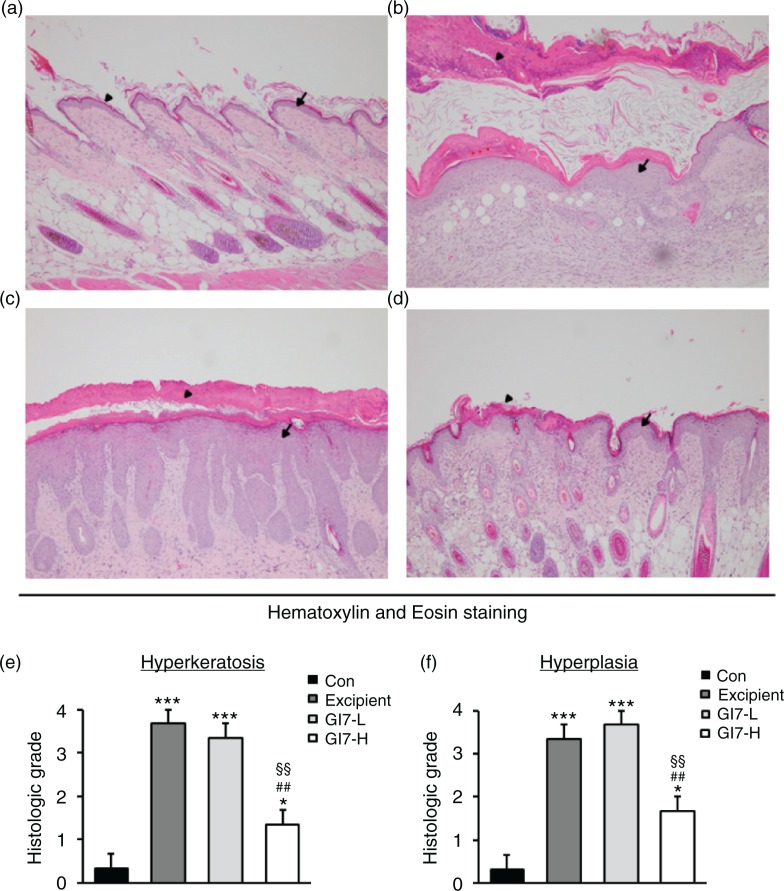
Histological changes of skin in NC/Nga mice. H&E-stained dorsal skin lesions were observed in the control (a), excipient (b), GI7-L (c), and GI7-H (d) groups. GI7-H is demonstrated to have an inhibitory effect on hyperkeratosis (arrowhead) (e) and hyperplasia (arrow) (f) of AD-like skin lesions compared with the excipient and GI7-L group. Results are expressed as the mean+SD (*n*=6). *p<0.05, ***p<0.001 versus the control group; ^#^ *p*<0.05, ^##^ *p*<0.01 versus the excipient group; ^§§^ *p*<0.01 versus the GI7-L group.

**Fig. 4:**
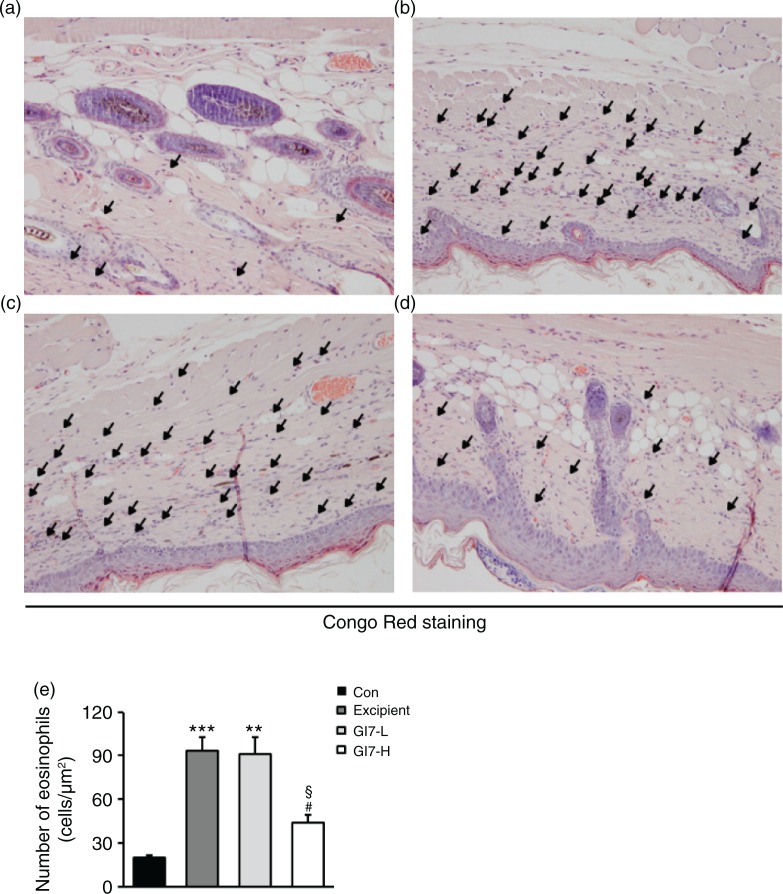
Eosinophil infiltration in the dorsal skin of mice. CR-stained dorsal skin lesions were observed in the control (a), excipient (b), GI7-L (c), and GI7-H (d) groups. GI7-H is demonstrated to have an inhibitory effect on eosinophil infiltration (arrow) of AD-like skin lesions compared with that of the excipient and GI7-L groups (e). Results are expressed as the mean+SD (*n*=6). **p<0.01, ***p<0.001 versus the control group; ^#^ *p*<0.05 versus the excipient group; ^§^ *p*<0.05 versus the GI7-L group.

**Fig. 5:**
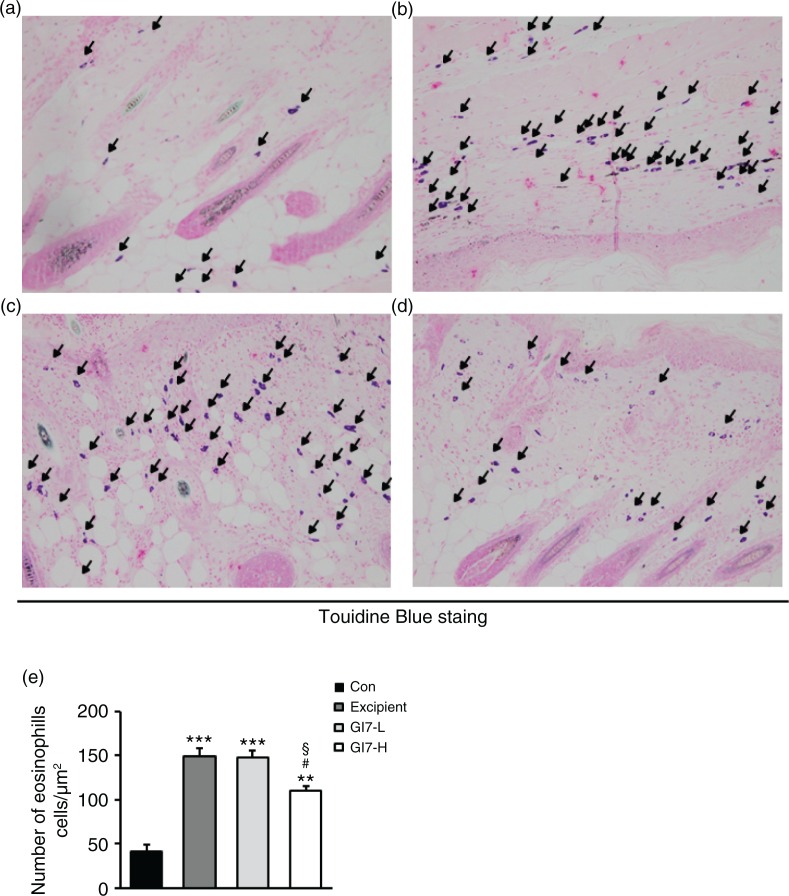
Mast cell infiltration in the dorsal skin of mice. TB-stained dorsal skin lesions were observed in control (a), excipient (b), GI7-L (c), and GI7-H (d) groups. GI7-H is demonstrated to have an inhibitory effect on mast cell infiltration (arrow) of AD-like skin lesions compared with that of the excipient and GI7-L groups (e). Results are expressed as the mean+SD (*n*=6). **p<0.01, ***p<0.001 versus the control group; ^#^ *p*<0.05 versus the excipient group; ^§^ *p*<0.05 versus the GI7-L group.

The severity of skin lesions in each group at week 12 is shown in Fig. 2a. At week 12, the dorsal skin of DNCB-treated NC/Nga mice exhibited severe erythema, erosion, and dryness. Among the treatment groups, there were markedly increased signs of AD-like skin lesions in the excipient group, such as bleeding, severe itching, and a rash. Administration of GI7-L (10^7^ CFU/day) did not reduce the histological grade of AD-like skin lesions, whereas GI7-H (10^9^ CFU/day) attenuated the development of AD-like skin lesions. The dermatitis score of AD-like skin lesions between the excipient and GI7-L groups was not significantly different throughout the 8 weeks of treatment (Fig. 2b). However, the GI7-H group showed a steady decrease in dermatitis score; this decrease began to be significantly different from week 9 compared with that of the excipient and GI7-L groups. The final AD index of the GI7-H group (4.4±0.7) was scored significantly lower than that of the excipient (7.7±0.4) and GI7-L (7.0±0.8) groups. The chronological profile of the scratching behavior observed in the control, excipient, GI7-L, and GI7-H groups is shown in Fig. 2c. The scratching behavior of the control group was determined to be 3.1±1.3; the score significantly increased in the excipient and GI7-L groups. By contrast, the scratching score of the GI7-H group (18.5±6.8) was significantly lower than those of the excipient (45±5) and GI7-L (42.6±4.2) groups. The spleen contains a range of immune cells and plays an important role in regulating the immune response. Because splenic enlargement or splenomegaly indicates abnormality of immune system function in AD (16, 17), we compared the change of morphological features of the spleen among the groups. The mice receiving excipient or GI7-L were found to have significantly enlarged spleens compared to those of the control group (*p*<0.001). However the spleen size was reduced in the mice treated with GI7-H (Fig. 2d). Meanwhile, at week 12, the dorsal skin of each mouse was prepared for examination. Thicker epidermis and dermis were observed in the excipient and GI7-L mice compared with that of the control mice (Fig. 3a–d), and these epidermal lesions included hyperkeratosis and hyperplasia with microabscess formation of the epithelium (Fig. 3e and f). However, hyperkeratosis and hyperplasia were significantly attenuated in the epidermal tissues of GI7-H–treated mice compared with those of the excipient group (*p*<0.01 and *p*<0.05, respectively) and the GI7-L group (*p*<0.01 and *p*<0.01, respectively). To investigate the effect of GI7-H on DNCB-induced infiltration of eosinophils and mast cells into skin lesions, the dorsal skin of each group was stained with CR and TB, respectively. The number of CR-stained eosinophils in skin lesions of the excipient group (93.3±9.1 cells) and the GI7-L group (90.6±12 cells) was significantly increased compared with that of the control group (20±1.5 cells) (Fig. 4a–e). Administration of GI7-H (43.6±5.6 cells) markedly lowered the number of eosinophils in the dorsal skin of DNCB-treated mice. Furthermore, the number of TB-stained mast cells in skin lesions of the excipient group (149±9.5 cells) and GI7-L group (147.3±8.1 cells) was significantly increased compared with the control group (41±8.5 cells) (Fig. 5a–e). Administration of GI7-H (110±5.1 cells) also significantly lowered the number of mast cells in the dorsal skin of DNCB-treated mice compared with the excipient and GI7-L groups.

### GI7 increases CD4^+^CD25^-^Foxp3^+^ Tregs and suppresses immune disorders

**Fig. 6:**
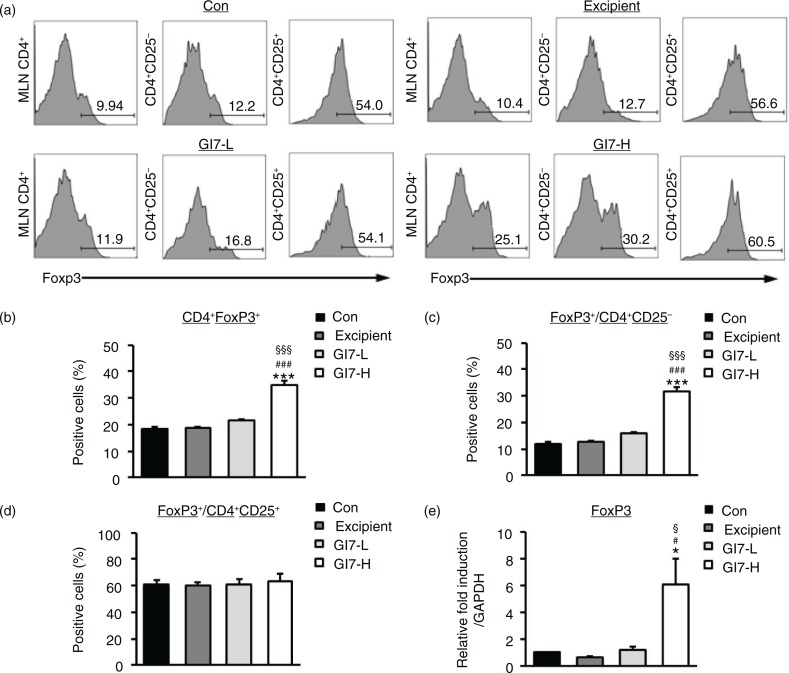
Expression of CD4^+^Foxp3^+^ T cells in MLNs of NC/Nga mice. (a) Foxp3 levels were examined by flow cytometry in MLN CD4^+^ T cells. Levels of Foxp3^+^ cells were analyzed in CD4^+^ (b), CD4^+^CD25^-^ (c), and CD4^+^CD25^+^ (d) populations. Expression level of Foxp3 in lesional skin was analyzed by qPCR. Relative fold induction of the target gene is compared with that of the housekeeping gene GAPDH. (e). Results are expressed as the mean+SD (*n*=6). *p<0.05, ***p<0.001 versus the control group; ^#^ *p*<0.05 versus the excipient group; ^§^ *p*<0.05, ^§§§^ *p*<0.001 versus the GI7-L group.

Tregs play an important role in various immune responses by modulating differentiation, proliferation, and function of various immune cells such as CD4^+^ T cells (11). Evidence of the importance of CD4^+^Foxp3^+^ Tregs was also shown through the observed immune modulation by probiotics in inflammatory disease models including AD (10). Therefore, CD4^+^Foxp3^+^ Tregs might play a critical role in the pathogenesis of AD. In this regard, we analyzed whether administration of GI7 affects the production of CD4^+^Foxp3^+^ Tregs. Tregs were isolated from MLNs, and the population of CD4^+^Foxp3^+^ T cells was measured by flow cytometry. As a result, administration of GI7-H was found to significantly increase the percentage of Foxp3-expressing CD4^+^ T cells (34.7±1.7%) compared to that of the control (18.3±0.9%), excipient (18.6±0.3%), and GI7-L (21.6±0.4%) groups (Fig. 6a and b). Increased Foxp3-expressing T cells originated from CD4^+^CD25^-^ Tregs rather than CD4^+^CD25^+^ Tregs (Fig. 6c and d). There was no significant difference in the CD4^+^CD25^+^Foxp3^+^ Tregs population among the treatment groups (Fig. 6d). We also confirmed that administration of GI7 significantly increased Foxp3 levels in the dorsal skin of DNCB-treated mice (Fig. 6e).

**Fig. 7:**
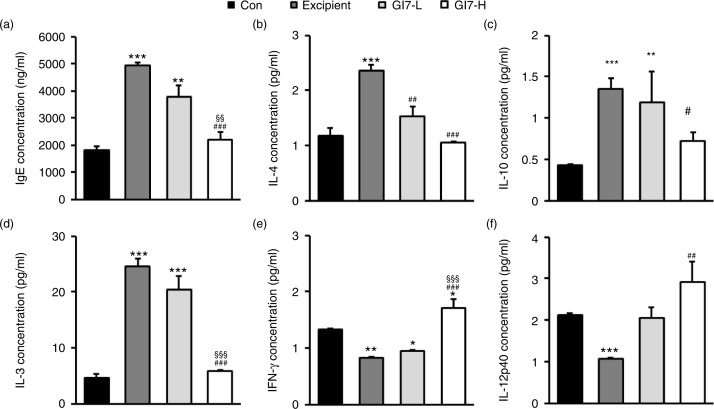
Serum IgE and inflammatory cytokine levels. Blood was obtained from NC/Nga mice at week 12 for which the IgE (a), IL-4 (b), IL-10 (c), IL-13 (d), IFN-γ (e), and IL-12p40 (f) serum levels were determined using ELISA. Results are expressed as the mean+SD (*n*=12). **p<0.01, ***p<0.001 versus the control group; ^###^ *p*<0.001 versus the excipient group; ^§§§^ *p*<0.001 versus the GI7-L group.

Next, we examined whether GI7-induced Foxp3 expression in Tregs has an immunomodulatory capacity and modulates the parameters for AD progression. First, because an increased IgE level is a representative indicator of AD, the levels of IgE in the serum were assessed. The IgE levels in the control group were determined to be roughly 1,816.4±150.9 ng/mL, whereas the excipient group showed a significantly elevated value of around 4,918.4±114.4 ng/mL (Fig. 7a). However, the serum IgE levels were decreased by administration of GI7, where the GI7-L and the GI7-H groups showed 3,791.7±396.9 and 2,207.8±252.8 ng/mL, respectively. Although the difference between the excipient and GI7-L groups was not significant (*p*>0.05), the IgE level in the GI7-H group was significantly lower than those in the GI7-L and excipient groups. Next, we measured the levels of IL-4, IL-10, and IL-13, all of which represent Th2 immune responses. The IL-4, IL-10, and IL-13 serum levels in the excipient group mice were determined to be 2.3±0.1, 1.3±0.1, and 24.5±1.4 pg/mL, respectively. In contrast, the levels of the respective Th2 cytokines in the GI7-H group were decreased to 1.0±0.1, 0.7±0.1, and 5.8±0.2 pg/mL, respectively, levels that were significantly different from the corresponding levels in the excipient group (Fig. 7b–d). There were no significant differences in the levels of IL-10 and IL-13 between the GI7-L and excipient groups, but the IL-4 level of the GI7-L group (1.53±0.1 pg/mL) was significantly lower than that of the excipient group. On the contrary, IFN-γ and IL-12p40 represent Th1 immune responses. The IFN-γ and IL-12p40 serum levels in the excipient group were determined to be 0.8±0.1 and 1.0±0.1 pg/mL, respectively, levels that were significantly lower than those in the control group. By contrast, the respective Th2 cytokines in the GI7-H group were increased to 1.7±0.2 and 2.9±0.5 pg/mL, levels that were significantly higher than those in the excipient group (Fig. 7e and f). The effect of GI7-L on the serum levels of IFN-γ and IL-12p40 were marginal, and no significant difference between the GI7-L and excipient groups was found.

### GI7 modulates Th2 immune responses and skin barrier– and antimicrobial-related gene expression

**Fig. 8:**
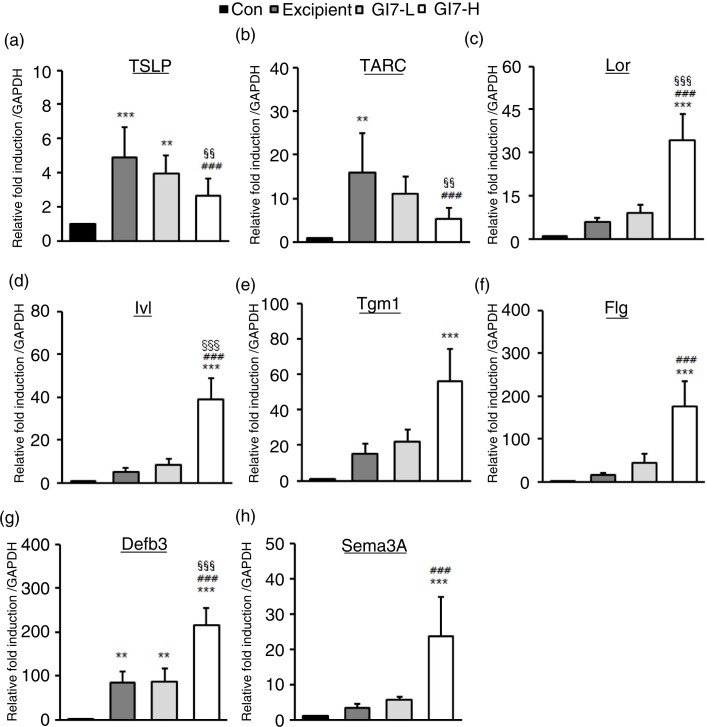
Skin barrier– and antimicrobial-related genes and chemokine expression in NC/Nga mice. Expression levels of TSLP (a), TARC (b), Lor (c), Ivl (d), Tgm1 (e), Flg (f), Defb3 (g), and Sema3A (h) in lesional skin was analyzed by qPCR. Relative fold induction of the target genes is compared with the housekeeping gene GAPDH. Results are expressed as the mean+SD (*n*=12). **p<0.01, ***p<0.001 versus the control group; ^###^ *p*<0.001 versus the excipient group; ^§§§^ *p*<0.001 versus the GI7-L group.

AD leads to an enhanced KC-driven stimulation of epidermal DC (18). Moreover, TSLP is one of the candidates for triggering Th2 commitment by activating DCs that induce differentiation of naïve T cells to Th2 cells, the expression of which is upregulated in AD-like skin lesions (19). TSLP from KCs induces the expression of Th2 chemokines TARC and macrophage-derived chemokine (MDC). The levels of TSLP (4.8±1.8-fold) and TARC (19.3±12.0-fold) in dorsal skin lesions of excipient mice were significantly increased compared with that of the control mice (Fig. 8a and b). However, GI7-H treatment significantly decreased both the levels of TSLP (2.6±0.6-fold) and TARC (8.9±3.7-fold) compared with the excipient and GI7-L (3.4±1.2- and 15.1±5.1-fold, respectively) mice. The production of MDC in the tissue was not significantly different among the treatment groups (data not shown). As shown in Fig. 8c–g, the levels of Lor (34.1±9.3-fold), Ivl (39.0±9.9-fold), Tgm1 (55.9±18.2-fold), Flg (176.1±18.2-fold), and antimicrobial peptides such as β-defensin 3 (Defb3) (214.7±40.4-fold) were significantly increased in the dorsal skin of GI7-H mice compared to that of the control mice. The expression of Defb2, a β-defensin, in the tissue was not significantly different among the treatment groups (data not shown). We also tested whether probiotics affect the mRNA level of semaphorin 3A (Sema3A), the regulatory factor of neuronal elongation. Sema3A plays an inhibitory role for C-fiber elongation in the upper layer of the epidermis, and decreased expression of Sema3A has been found in lesional skin of AD (20). In this study, the level of Sema3A in the dorsal skin lesions of GI7-H mice was significantly increased compared with that of the control (23.5±11.2-fold) and excipient (3.2±1.1-fold) groups (Fig. 8h).

### Changes in fecal bacteria

Finally, we quantified the seven species of GI7 and total bacteria in fecal samples collected from the respective groups at the end of the study by qPCR with species- or group-specific primer sets. As summarized in Table 3, the levels of total bacteria were constant among the groups. Compared with the control group, however, the excipient group showed significant decreases in the levels of all the targeted species except *B. breve* (*p*<0.001). The administration of GI7 significantly increased the levels of the seven species compared with that of the excipient group. As expected, the impact of GI7-H on the fecal bacteria seemed to be greater than GI7-L, resulting in significant differences between the two groups in the levels of all the target species.

**Table 3:** Quantification of fecal bacteria

	Control	Excipient	GI7-L	GI7-H
	Mean±SD	Mean±SD	Mean±SD	Mean±SD
*Lactobacillus acidophilus*	8.7±0.10	4.23±0.15***	8.61±0.03###	9.73±0.04***###§§§
*Lactobacillus rhamnosus*	9.78±0.04	6.70±0.23***	9.7±0.04##	10.41±0.05***###§§§
*Lactobacillus plantarum*	10.61±0.16	7.85±0.28***	11.03±0.42###	11.71±0.22**###
*Bifidobacterium bifidum*	8.92±0.08	4.63±1.35***	9.20±0.01###	9.70±0.03###
*Bifidobacterium breve*	8.58±0.38	8.25±0.22	8.96±0.02^#^	9.76±0.05***###§
*Lactococcus lactis*	6.89±0.37	3.08±0.71***	6.90±0.24###	10.14±0.13***###§§§
*Streptococcus thermophilus*	9.74±0.69	7.38±0.28***	10.05±0.43###	11.17±0.16*###
Total bacteria	12.82±0.02	12.83±0.01	12.85±0.02	12.90±0.06

Levels of the bacteria species and total bacteria were analyzed by qPCR. Bacterial groups were quantified for fecal samples at week 12. Bacterial quantities were expressed as log_10_ (bacterial cells/g of stool). Results are expressed as the mean±SD (*n*=12).

* *p*<0.05,

** *p*<0.01,

*** *p*<0.001 versus the control group

## *p*<0.01,

### *p*<0.001 versus the excipient group

§ *p*<0.05,

§§§ *p*<0.001 versus the GI7-L group.

## Discussion

**Fig. 9:**
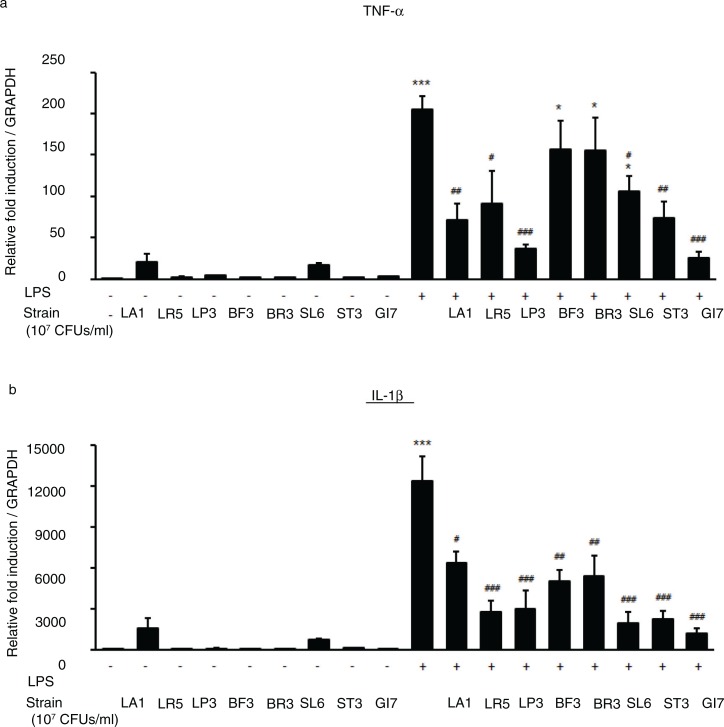
Expression levels of TLR4-mediated proinflammatory cytokines TNF-α and IL-1β in RAW 264.7 cells cotreated with LPS and GI7 strains. Cells were treated 15 ng/mL LPS for 6 h with or without LA1, LR5, LP3, BF3, SL6, ST3, or GI7 (10^7^ CFU/mL). Results are expressed as the mean+SD (*n*=6). *p<0.05, ***p<0.001 versus the control group (0 ng/mL LPS-treated cells); ^#^ *p*<0.05, ^##^ *p*<0.01, ^###^ *p*<0.001 versus the LPS-treated group.

Despite numerous studies on allergies, the protective effects of probiotics have been minimally demonstrated in humans because of limitations such as duration of ingestion and short follow-up period (21–23). Previously, we identified the probiotic formulation GI7 that has potent anti-inflammatory properties, and we studied the protective effect of GI7 in a mouse colitis model in which the underlying mechanism involved modulation of proinflammatory cytokine production (Kim et al., manuscript in preparation). In this study, we also confirmed the potent anti-inflammatory effects of GI7 following *in vitro* functional studies on the reduction of tumor necrosis factor-α and IL-1β expression in LPS-treated RAW 264.7 cells (Fig. 9). These findings suggest that the GI7 mixture might be an applicable treatment of immune disorders. Hence, here we investigated a mouse AD model, another model of inflammatory disease, and sought to confirm the therapeutic effects and a novel mechanism of GI7. Tregs are known to modulate the upregulation of Th1 and Th2, thereby inhibiting the progression of inflammation in allergic conditions (24). Many studies have suggested the involvement of Tregs in controlling various aspects of AD (25–27). Ingested probiotics are recognized by pattern recognition receptors, such as TLRs on DCs (28), that are essential for immunological homeostasis in the gut and that play an important role in allergic diseases; therefore, CD4^+^Foxp3^+^ Tregs are increased in MLNs (10, 27). In this study, analysis of Tregs in MLNs by using flow cytometry showed that the percentage of CD4^+^Foxp3^+^ Tregs increased significantly in the GI7-H group compared with that of the control group, whereas no changes were observed in the control and GI7-L groups (Fig. 6). Thus, the result indicates that the high dose of GI7-H has the potential to increase CD4^+^Foxp3^+^ Tregs. The Th1 and Th2 reaction types can reciprocally regulate one another, and modulation of immunological imbalance can be the target of AD therapy (1). Several reports have shown inhibition of the development of AD-like skin lesions in NC/Nga mice by decreasing the Th2 response (29–31). The immunomodulatory activities of GI7 were achieved by not only suppressing the expression of Th2 type inflammatory cytokines and chemokines such as IL-4, IL-10, IL-13, TSLP, and TARC, but also by increasing Th1 type immunosuppressive cytokines such as IFN-γ and IL-12p40. In particular, Th2 cytokines are known to be triggered by TSLP and TARC produced by KCs (5, 6). KC differentiation genes such as *Lor*, *Ivl*, *Tgm1*, *Flg*, and *Defb* play key roles in facilitating terminal differentiation of the epidermis and formation of the skin barrier (8, 32), the expression levels of which were found to be modulated by GI7. These observations could be an outcome of the enrichment of CD4^+^Foxp3^+^ Tregs by GI7. Interestingly, Kwon et al. (10) reported that experimental IBD and AD mice exhibited reduced numbers of CD4^+^Foxp3^+^ Tregs in inflamed sites compared with the normal mice. In general, severe enteropathy develops in humans deficient in Foxp3 expression and mice deficient in Tregs (33–35), and Foxp3^+^ Tregs of psoriasis patients are susceptible to differentiation into IL-17A that has a highly pathogenic role in skin inflammation and the development of psoriatic plaques (36–38). Although both Foxp3^high^- and Foxp3^low^-expressing Tregs produce IL-17A, more cells produce IL-17A within the Foxp3^low^ population (39). Thus, CD4^+^Foxp3^+^ Tregs can have a particularly important role in preventing inflammatory disease. This supports our finding that the reduced AD-like symptoms by GI7 are partially due to Foxp3 expression.

Little information is available regarding the mechanism by which probiotic treatment increases CD4^+^Foxp3^+^ cells. However, a study has shown that a probiotic formulation including five bacterial strains was capable of inducing DCs to promote the generation of CD4^+^Foxp3^+^ Tregs in MLN (10). Regulatory DCs (rDCs) generated by the multistrain formulation converted CD4^+^CD25^-^ T cells into CD4^+^CD25^-^Foxp3^+^ T cells. They also generated CD11c^+^ DCs that expressed markers of rDC including IL-10, transforming growth factor (TGF)-β, cyclooxygenase (COX)-2, and indoleamine 2,3-dioxygenase (iDO). The addition of inhibitors of TGF-β, COX-2, or iDO also strongly suppressed the generation of Foxp3^+^ Tregs. In this respect, the present study demonstrated that GI7 also has immunomodulatory activity by which CD4^+^Foxp3^+^ Tregs was induced. In addition, we identified that administration of GI7 increased Foxp3 in the CD4^+^CD25^-^ population instead of the CD4^+^CD25+ population (Fig. 6). Although Foxp3 expression was initially known to be restricted to the CD4^+^CD25^+^ Tregs population, Nishioka et al. reported that a population of Foxp3^+^CD4^+^CD25^-^ Tregs was present in aged mice (40). In addition, recent reports have shown that Foxp3^+^CD4^+^CD25^-^ Tregs confer a regulatory function on T cells (41, 42). Collectively, the finding that Foxp3 expression could be independent of CD25 expression would be a novel phenomenon for the immunomodulatory actions of probiotics.

In conclusion, the combined probiotics in GI7 played an important role in the suppression of AD-like symptoms in DNCB-treated NC/Nga mice, through the upregulation of CD4^+^Foxp3^+^ Tregs. Administration of GI7 resulted in decreased Th2-mediated cytokines and increased Th1-mediated cytokines. In addition, the expression of antimicrobial, skin barrier–related genes or chemokines was effectively modulated by GI7 treatment. Our findings demonstrate that generation of CD4^+^Foxp3^+^ Tregs capable of mediating immune suppression in response to GI7 may have a therapeutic role in the treatment of AD. Further studies are necessary to consider aspects such as the mechanism involved in the upregulation of CD4^+^Foxp3^+^ Tregs by GI7 and other protective roles of GI7 in AD. Moreover, more research needs to be conducted regarding the efficacy of GI7 in promoting human health.
